# SOX2-RNAi attenuates S-phase entry and induces RhoA-dependent switch to protease-independent amoeboid migration in human glioma cells

**DOI:** 10.1186/1476-4598-10-137

**Published:** 2011-11-09

**Authors:** Felix Oppel, Nadja Müller, Gabriele Schackert, Sandy Hendruschk, Daniel Martin, Kathrin D Geiger, Achim Temme

**Affiliations:** 1 Department of Neurosurgery, Section Experimental Neurosurgery and Tumor Immunology, University Hospital Carl Gustav Carus, TU Dresden, Dresden, Fetscherstr. 74, 01307 Dresden, Germany; 2 Department of Neuropathology, Institute of Pathology, Medical Faculty Carl Gustav, TU Dresden, Fetscherstr. 74, 01307 Dresden, Germany

## Abstract

**Background:**

SOX2, a high mobility group (HMG)-box containing transcription factor, is a key regulator during development of the nervous system and a persistent marker of neural stem cells. Recent studies suggested a role of SOX2 in tumor progression. In our previous work we detected SOX2 in glioma cells and glioblastoma specimens. Herein, we aim to explore the role of SOX2 for glioma malignancy in particular its role in cell proliferation and migration.

**Methods:**

Retroviral shRNA-vectors were utilized to stably knockdown SOX2 in U343-MG and U373-MG cells. The resulting phenotype was investigated by Western blot, migration/invasion assays, RhoA G-LISA, time lapse video imaging, and orthotopic xenograft experiments.

**Results:**

SOX2 depletion results in pleiotropic effects including attenuated cell proliferation caused by decreased levels of cyclinD1. Also an increased TCF/LEF-signaling and concomitant decrease in Oct4 and Nestin expression was noted. Furthermore, down-regulation of focal adhesion kinase (FAK) signaling and of downstream proteins such as HEF1/NEDD9, matrix metalloproteinases pro-MMP-1 and -2 impaired invasive proteolysis-dependent migration. Yet, cells with knockdown of SOX2 switched to a RhoA-dependent amoeboid-like migration mode which could be blocked by the ROCK inhibitor Y27632 downstream of RhoA-signaling. Orthotopic xenograft experiments revealed a higher tumorigenicity of U343-MG glioma cells transduced with shRNA targeting SOX2 which was characterized by increased dissemination of glioma cells.

**Conclusion:**

Our findings suggest that SOX2 plays a role in the maintenance of a less differentiated glioma cell phenotype. In addition, the results indicate a critical role of SOX2 in adhesion and migration of malignant gliomas.

## Background

Despite multimodal treatment the prognosis for glioblastoma (GBM), the most common and most malignant brain tumor remains poor, with the majority of patients dying within 1 year after diagnosis [1]. Glioblastomas, gliomas of WHO grade IV, diffusely spread into the surrounding brain and the invading tumor cells migrate along the white matter tracks and assemble satellites around neuron cell bodies, blood vessels and the subpial region [2,3]. Since glioblastoma cells infiltrate wide areas of the brain every resection of the bulk tumor is usually followed by a tumor re-initiation at the resection site or at another place in the brain [4,5]. The cellular origin of glioblastoma is still under investigation and it is hypothesized that this tumor arises from transformed pluripotent precursor cells, so called glioblastoma initiating stem cells [6,7].

Recently, we reported expression of the stem cell marker SOX2 in glioblastoma specimens [8]. SOX2 is a 34 kDa HMG-box containing transcription factor belonging to the sex determining region Y (SRY)-box proteins which play an important role in development, in particular in the central nervous system [9-11]. It has been reported, that SOX2 preserves the undifferentiated state of neural progenitor cells in chicken [12] and is a persistent marker of multipotent neural stem cells, both in murine embryos and mice [13]. SOX2 positively controls self-renewal of neural stem cells [14] and its ectopic overexpression inhibits neural fate differentiation [13,15,16]. Several lines of evidence suggest an oncogenic role of SOX2 in tumor progression. Hence SOX2 expression was found to be a negative prognostic marker of esophageal squamous cell carcinoma [17], and was correlated with later stages and invasive phenotype of pancreatic carcinoma [18]. Furthermore, SOX2 has been observed in 43% of basal breast carcinomas which was associated with a less differentiated phenotype [19]. Contrary to these reports SOX2 overexpression and concomitant Oct4 overexpression was found to be a marker of less progressive squamous cell lung cancer and hypopharyngeal squamous cell carcinoma, respectively, and predicted a better clinical outcome [20,21]. The situation becomes even more complicated by a recent report describing a tumor-suppressive function of SOX2 in gastric cancers and gastric cancer cell lines [22].

A recent study revealed robust SOX2 expression in brain tumors of glial lineages expressing the astrocytic marker protein glial fibrillary acidic protein (GFAP) [23]. Other brain tumors, such as medulloblastomas and pineoblastomas, displayed markers of neuronal differentiation and lacked SOX2 expression [23]. It can be assumed that SOX2 in gliomas might augment the maintenance of a less differentiated astroglial phenotype and positively influence proliferation of glioblastoma cells in a similar manner as in glial progenitor cells. Therefore, SOX2 might represent a suitable target for RNAi to treat malignant gliomas. In line with this, it has recently been reported that RNAi-mediated knockdown of SOX2 in glioblastoma tumor initiating cells (TICs) led to impaired proliferation [24]. However, the molecular mechanisms leading to this decreased cell growth remain obscure. So far only a small number of investigations have touched the role of SOX2 in governing cancer cell proliferation and migration capacity on the molecular level [25,26].

In the present study we sought to elucidate the role of SOX2 in glioma malignancy. We also wanted to investigate whether RNAi of SOX2 might be amendable to treat malignant gliomas. For the investigations we used SOX2-positive cell lines U343-MG and U373-MG, which upon RNAi of SOX2 were still viable but showed an attenuated S-phase entry associated with decreased levels of phosphorylated RB protein and reduced levels of cyclinD1. Notably, the cells lost expression of Oct4 and Nestin indicative for a shift from a stem cell-like phenotype to a more differentiated cell type. Furthermore, a loss of invasive proteolysis-dependent migration capacity and a reduced matrix adhesion due to a reorganized actin cytoskeleton were noted. These changes were accompanied by an increased TCF/LEF- and diminished FAK-signaling. Further analysis revealed that the loss of invasive proteolysis-dependent migration capacity of glioma cells with knockdown of SOX2 could be compensated by a shift to amoeboid migration governed by increased RhoA/ROCK2 signaling. In line with this, xenograft experiments revealed an increased dissemination of U343-MG cells transduced with shRNA targeting SOX2 in mouse brain which was associated with a decreased survival of mice. Our study is the first that describes the impact of SOX2-RNAi on the cell cycle and on the migratory behavior of glioma cells on the molecular and cellular level. In particular, a potential development of an amoeboid phenotype after SOX2-RNAi might be dangerous when SOX2 or downstream molecules are targeted in cancer therapies.

## Results

### SOX2-RNAi induces morphological changes in U343-MG and U373-MG cells and an attenuated cell growth

**Figure 1:**
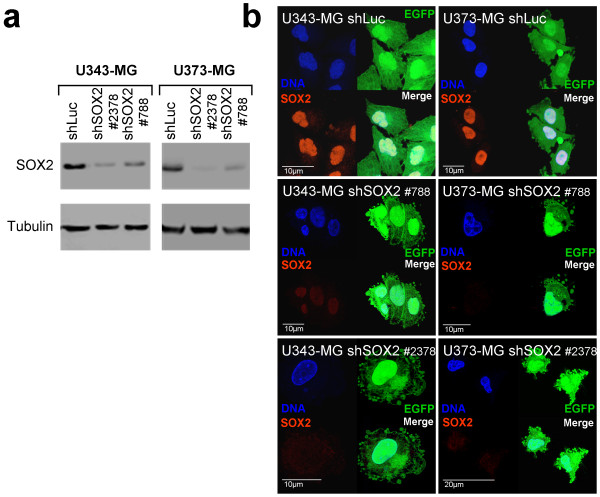
**Efficient transduction and stable knockdown of SOX2 in glioblastoma cell lines U343-MG and U373-MG**. **a: **Western blot analysis showing knockdown of SOX2 in transduced U343-MG and U373-MG cells using pRVH-N1-EGFP vectors encoding the small hairpins sh788 and sh2378. As control, total protein lysates of cells transduced with shLuc are included. The membrane was stripped and probed for tubulin in order to confirm equal protein loading. **b: **Indirect immunofluorescence analysis of SOX2 expression in U343-MG and U373-MG cells after transduction of retroviral vectors encoding shRNA-shSOX2-788 and shRNA-SOX2-2378. Note the loss of SOX2-immunosignals in transduced EGFP-positive cells. Controls transduced with shLuc are included.

U343-MG and U373-MG cells were tranduced using retroviral vectors encoding for EGFP and for small hairpins 788 or 2378 (shSOX2 #788 or shSOX2 #2378), respectively, targeting SOX2 mRNA. As control a retroviral vector encoding a shRNA targeting luciferase was included. Both SOX2-shRNAs efficiently blocked SOX2 protein expression when tested in Western blot analysis and indirect immunofluorescence analysis using an antibody specific for SOX2 and when compared to the appropriate control cells (Figure 1a, b).

**Figure 2:**
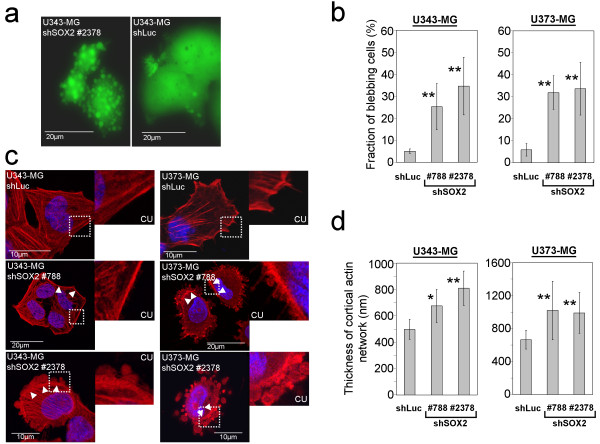
**Development of non-apoptotic membrane protrusions in SOX2-silenced U343-MG and U373-MG cells**. **a: **Representative microscopy images showing U343-MG cells with knockdown of SOX2 and control cells transduced with shLuc. **b: **Quantification of the fraction of cells showing membrane blebs after knockdown of SOX2. Bars indicate standard deviation. **c: **Confocal laser scan micrsocopy images showing Phalloidin-TRITC staining of actin filaments in U343-MG and U373-MG cells after knockdown of SOX2. CU: Close ups on cortical actin filaments. As control shLuc transduced U343-MG and U373-MG cells are included. Note the increased thickness of cortical actin filaments (arrowheads) and the development of membrane alterations in cells with knockdown of SOX2. **d:**. Analysis of the thickness of cortical actin filaments: The thickness of the cortical actin network is increased in cells with knockdown of SOX2 when compared to U343-MG and U373-MG shLuc control cells. * p < 0.05; **p > 0.01 when SOX2-silenced cell were compared to shLuc controls.

The shRNA-treated cells showed strong EGFP expression, marking successful gene transfer, within two days after transduction (see additional file 1). Starting with day 4 after transduction significant fractions of glioma cells expressing shSOX2 #788 and #2378 started to develop membrane alterations, reminiscent of apoptotic blebs (Figure 2a, b). Furthermore, these cells tend to lose adherence from the cell culture dish. Phalloidin-TRITC-staining of the actin filaments of cells with knockdown of SOX2 frequently revealed a reorganized actin cytoskeleton displaying a significantly thickened cortical actin network with emerging membrane protrusions containing actin filaments (Figure 2c, d).

**Figure 3:**
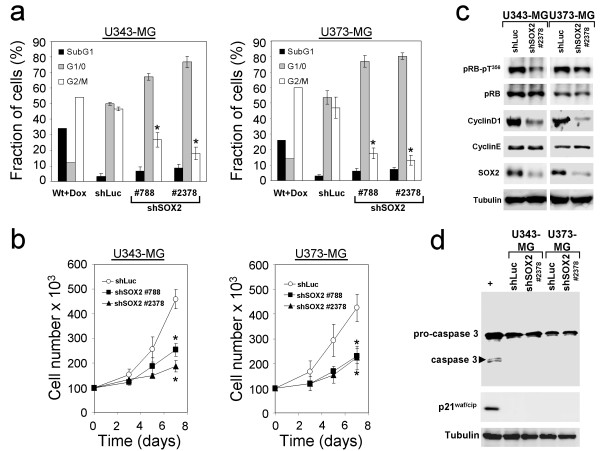
**Effects of SOX2-RNAi on cell proliferation, cell cycle and induction of apopotosis in U343-MG and U373-MG glioma cells**. **a: **Propidium iodide-staining and cell cycle analysis of U343-MG and of U373-MG cells 5 days after transduction with shRNA vectors revealed no induction of apoptosis (SubG1 fraction) but a decrease of cell fractions in S/G_2_/M-phases. As controls, shLuc transduced cells were included. As positive control for apoptosis U343-MG and U373-MG wild type cells were treated with doxorubicin to induce apoptosis (Wt+Dox). **b: **Growth kinetics of U343-MG and U373-MG cells transduced with shSOX2 #788, shSOX2 #2378, and shLuc (control). Note the gradual decrease in cell numbers in glioma cells with knockdown of SOX2. Bars in **a: **and **b: **indicate standard deviation. * p < 0.05 when SOX2-silenced cell were compared to shLuc controls. **c: **Western blot analysis of SOX2 and of cell cycle regulator proteins cyclinD1, cyclinE, retinoblastoma protein (pRB) and of pRB-pT^356^, indicating phosphorylation by the cyclinD1-cdk4 complex. Note the decreases in cyclinD1 expression and the concomitant diminished levels of pRB-pT^356 ^in cells with knockdown of SOX2. **d: **Western blot analysis showing no cleavage of pro-caspase 3 and no increase of p21^waf/cip ^inhibitor of cyclin-dependent kinases Cdk4/6 in U343-MG and U373-MG cells transduced with shSOX2 #2378 and in controls transduced with shLuc. (+) depicts U343-MG cells treated with doxorubicin serving as positive control for induction of apoptosis and induction of p21^waf/cip^. Equal loading of lanes in **c: **and **d: **was verified using tubulin antibody.

Despite the appearance of these apoptosis-like membrane alterations no increases in the fraction of apoptotic cells having hypodiploid (SubG1) genomes were noted (Figure 3a). Instead, knockdown of SOX2 in U343-MG and U373-MG resulted in significant increases in the fraction of cells in G_1_-phase when compared to shLuc-transduced control cells (Figure 3a). Thus, knockdown of SOX2 obviously impaired the G_1 _to S-phase transition of glioma cells. Moreover, U343-MG and U373-MG cells with knockdown of SOX2 showed a decrease in cell growth when compared to the shLuc-treated controls (Figure 3b). However, it cannot completely be ruled out that this effect in part was due to the loss of detached cells with knockdown of SOX2 during standard cell cultivation. Next, the molecular mechanism underlying the diminished S-phase entry induced by loss of SOX2 function was examined. Western blot analysis five days after transduction revealed an impaired expression of cyclinD1 in U343-MG and U373-MG glioma cells with knockdown of SOX2 when compared to cells transduced with the shLuc control vector, whereas cyclinE levels remained unaffected (Figure 3c). Interestingly, a reduced level of retinoblastoma protein (RB protein) phosphorylated at threonine 356, one of the phosphorylation sites of the cyclinD1-Cdk4 complex, was noted (Figure 3c). On the other hand, a cell cycle arrest in G_1 _upon SOX2-RNAi through induction of cell cycle inhibitor p21^waf/cip ^was not detectable in the U343-MG cell line which is known to have functional p53 [27] and also no p21^waf/cip ^induction was observed in total protein lysates of U373-MG cells which have mutant p53 [28] (Figure 3d). Testing apoptosis by investigating the appearance of cleaved pro-caspase 3 in Western blot analyses showed no induction of apoptosis (Figure 3d). In addition, a clonogenic assay revealed only a moderate decrease in long term survival of cells with knockdown of SOX2 when compared to shLuc-control cells (see additional file 1). Thus, the prevailing effect of the SOX2 knockdown was an attenuated cell cycle progression which was accompanied by a notable change in cell adherence and cell morphology.

### SOX2 knockdown results in increased TCF/LEF-1-signaling and a decrease in expression of stem cell markers Oct4 and Nestin

**Figure 4:**
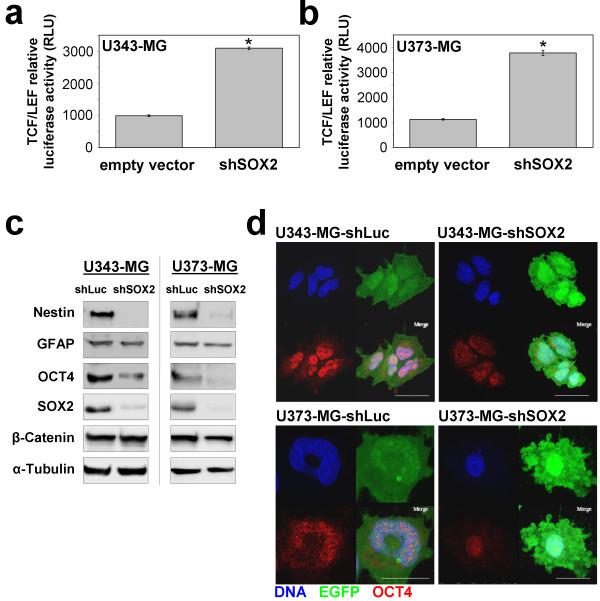
**SOX2-RNAi increases TCF/LEF-signaling and decreases Nestin and OCT4 steady state protein expression levels**. **a: **U343-MG and **b: **U373-MG glioma cells stably expressing TCF/LEF-driven luciferase were transduced with sh2378 or empty vector and analyzed. Note the increase in luciferase activity after knockdown of SOX2 in both glioma cell lines. Bars indicate standard deviation. * p < 0.05 when SOX2-silenced cell were compared to controls. **c: **Western blot analysis for stem cell markers Nestin and Oct4 in U343-MG and U373-MG cells with knockdown of SOX2 showing loss of protein expression when compared to shLuc controls. Steady state protein expression levels of astrocyte marker glial fibrillary acidic protein (GFAP) and β-Catenin remained unchanged. Equal loading of lanes was confirmed using tubulin antibodies. **d: **Indirect immunofluorescence showing decrease of Oct4 immunosignals in U343-MG and U373-MG cells treated with sh2378 when compared to shLuc controls. Magnification bars: 20 μm.

Next we were interested whether knockdown of SOX2 affects the differentiation status of the glioma cells besides the attenuated S-phase entry. Recent studies have demonstrated involvement of SOX2 in the wnt/β-Catenin-mediated regulation of Transcription factor T cell specific/Lymphoid enhancer binding factor (TCF/LEF) transcription factors. In particular, it has been shown, that SOX2 inhibits canonical wnt-signaling in vertebrates leading to decreased TCF/LEF-mediated transcriptional activity which concomitantly inhibited cellular differentiation processes [29]. When we investigated the β-Catenin/TCF/LEF transcriptional activity by using a firefly luciferase reporter gene assay we revealed a 3-fold and 4-fold higher TCF/LEF signaling in U343-MG and U373-MG cells with knockdown of SOX2 when compared to controls (Figure 4a, b). In parallel, Western blot and indirect immunofluorescence analyses demonstrated a decrease in the steady state expression levels of stem cell transcription factor Oct4 (Figure 4c, d). Also a decrease in Nestin expression was noted in U343-MG and U373-MG cells with knockdown of SOX2 whereas expression levels of the astrocytic marker GFAP and of β-catenin remained unchanged (Figure 4c). Our data suggest that SOX2 expression preserves stem cell characteristics and on the other hand can modulate the proliferation of U343-MG and U373-MG glioma cells.

### SOX2-RNAi decreases invasive proteolysis-dependent migration of glioma cells

**Figure 5:**
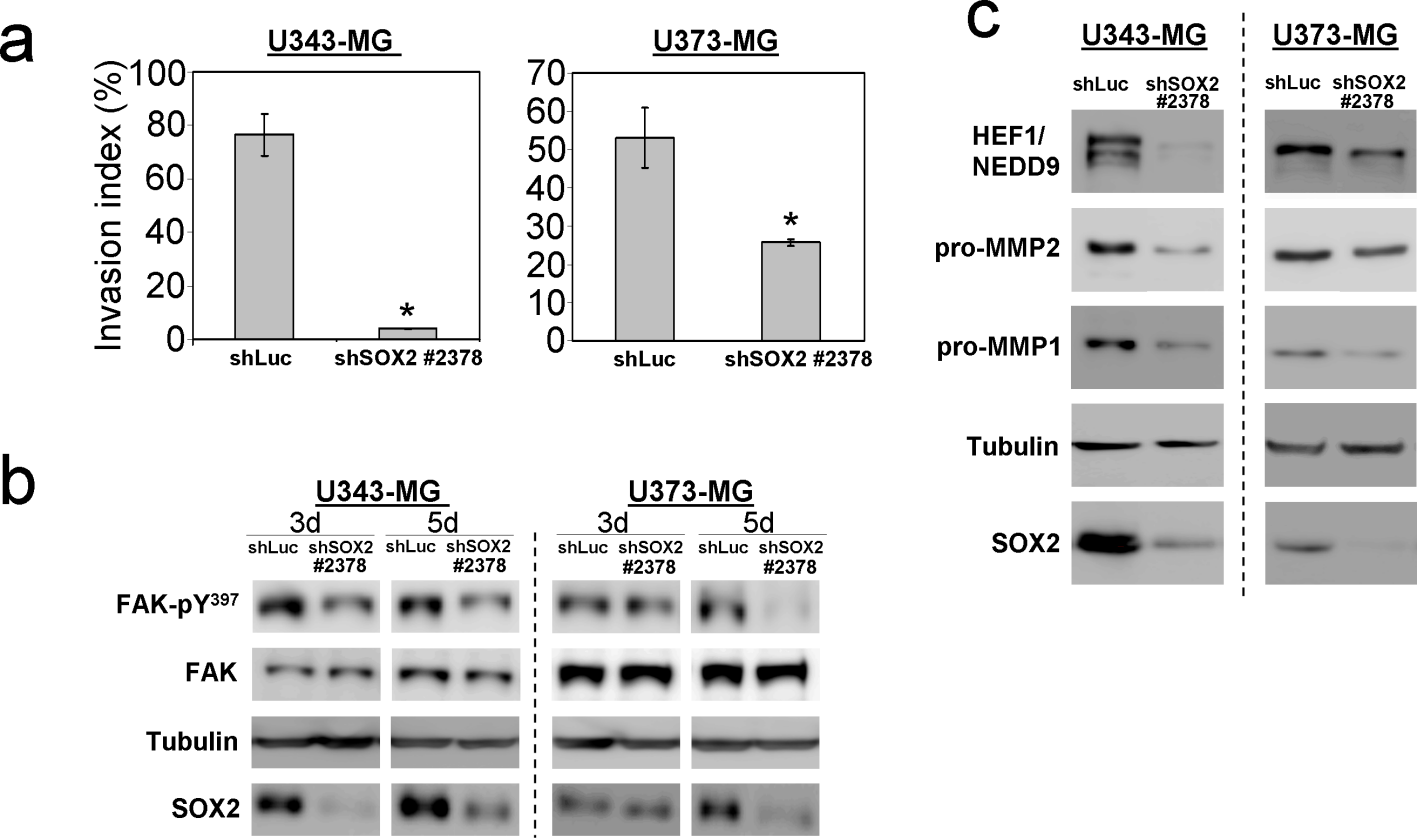
**SOX2-RNAi affects adhesion, invasion and migration of glioma cells**. **a: **Trans-well assay measuring the invasion of U343-MG- and U373-MG glioma cells with stable knockdown of SOX2 and of shLuc control cells using extra cellular matrix (ECM)-coated Boyden chambers. The calculation of the proteolysis-dependent invasion index was performed as described in the material and methods section. Bars indicate standard deviation. * p < 0.01 when cells with knockdown of SOX2 were compared to shLuc controls. **b: **Western blot analysis of focal adhesion kinase (FAK) and its activated phosphorylated form (FAK-pY^397^). FAK and FAK-pY^397 ^was analyzed in SOX2 silenced (shSOX2-2378) and in control cells (shLuc) at two different time points 3 days (3 d) and 5 days (5 d) after transduction. Note the decrease in FAK-pY^397 ^in cells with knockdown of SOX2. **c: **Western blot analysis showing diminished expression of the human enhancer of filamentation protein (HEF1/NEDD9), and of pro-matrix metalloproteinases (pro-MMPs) 1 and 2, in U343-MG and U373-MG glioma cells with knockdown of SOX2.

Because we observed remarkable morphological changes and a remodeling of the actin cytoskeleton of the glioma cells with knockdown of SOX2 we hypothesized that the migratory capacity of these cells might be affected. We therefore employed transwell migration assays to examine the invasive proteolysis-dependent migration capacity of U343-MG and U373-MG glioma cells through a membrane-bound collagen matrix. Interestingly, the proteolysis-dependent invasion potential of SOX2 depleted cells was significantly reduced compared to control cells (Figure 5a). The shSOX2-U373-MG cells had an invasion index of 25.7% (+/- 0.9%) compared to 53% (+/- 7.8%) measured for shLuc control cells. This effect was even stronger for U343-MG cells. Here, the invasion index was reduced from 76.5% (+/- 7.83%) for shLuc controls to 4.1% (+/- 0.22%) measured for the shSOX2-U343-MG cells. Thus, knockdown of SOX2 expression impaired proteolytic invasion of U343-MG and U373-MG glioma cells through collagen matrices. To elucidate, in which way the migratory behavior was compromised in the SOX2-knockdown cells, we focused on focal adhesion kinase (FAK), on its downstream signaling protein human enhancer of filamentation (HEF1/NEDD9) and metallo-matrixproteases which are essential for a proteolysis-dependent invasion and migration of glioma cells [30,31]. Initial step for FAK signaling is an autophosphorylation at Y397, which occurs after binding of integrins to the ECM. This autophosphorylation of FAK has been reported to promote the binding and activation of the proto-oncogenic cytosolic tyrosine kinase c-Src [32]. c-Src subsequently phosphorylates Crk-associated substrates (CAS) proteins, such as HEF1/NEDD9 [31]. Phosphorylated CAS proteins promote the activation of small GTPases such as cdc42 and Rac1 which affects membrane protrusion and migration [30]. Interestingly, Western blot analyses at two different time points 3 and 5 days after transduction revealed a marked reduction of FAK phosphorylation at Y397 for the SOX2-depleted glioma cells when compared to shLuc-transduced controls (Figure 5b). Moreover, our analyses revealed a downregulation of HEF1/NEDD9 and of metallo-matrixproteases pro-MMP1 and pro-MMP2 in SOX2-depleted U343-MG and U373-MG glioblastoma cells (Figure 5c). Thus, knockdown of SOX2 decreases expression levels for proteins involved in invasive proteolysis-dependent migration.

### Glioma cells with knockdown of SOX2 acquire an amoeboid-like phenotype by increased RhoA/ROCK2-signaling

**Figure 6:**
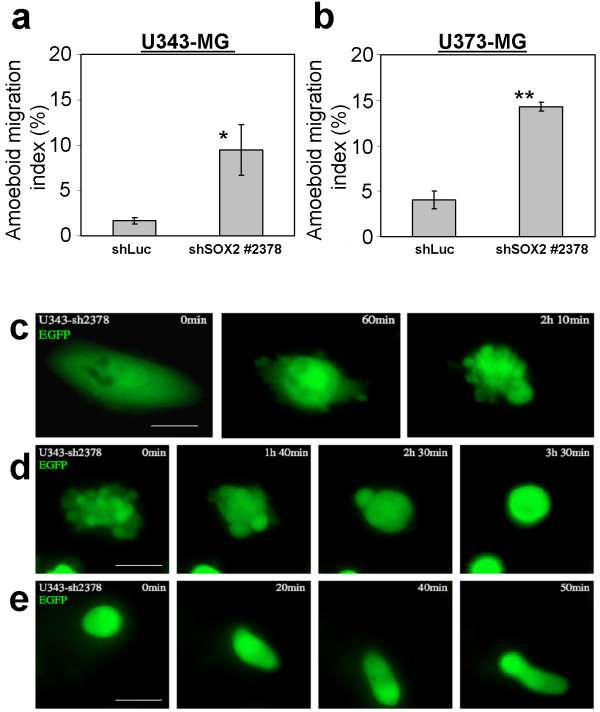
**SOX2-RNAi induces morphological changes in glioma cells**. Trans well assay using membranes lacking the ECM for determination of proteolysis-independent amoeboid-like migration of **a: **U343-MG and **b: **U373-MG cells with knock down of SOX2. The calculation of the amoeboid-like migration index was performed as described in the material and methods section. Bars indicate standard deviation. * p < 0.05; ** p < 0.01 when cells with knockdown of SOX2 were compared to shLuc controls. **c-e: **U343-MG cell transduced with shSOX2 #2378 and expressing the marker EGFP were monitored by life cell imaging. **c: **An adherent cell with normal cell morphology develops protrusions of the membrane which appear as little blebs. **d: **An intensive blebbing cell develops less frequent, but larger blebs, and acquire a round cell morphology; **e: **A fully rounded cell contracts the cell body and migrates. Magnification bars: 30 μm.

When we tested migration through uncoated membranes we noted a significant higher number of U343-MG and U373-MG glioma cells with knockdown of SOX2 in the bottom well of the Boyden-chamber bearing a rounded cell morphology and having membrane protrusions when compared to cells transduced with the shLuc control vector. We hypothesized that these cells compensate the loss of invasive proteolysis-dependent migration by acquiring an amoeboid-like migration. This kind of cell motility has been described to use a path-finding instead of a path generating migration strategy [33]. We calculated that the shSOX2-U343-MG cells had an amoeboid migration index of 9.47% (+/- 2.77%) compared to 1.67% (+/- 0.34%) measured for shLuc control cells (Figure 6a). The amoeboid migration index for U373 was 14.3% (+/- 0.5%) and for shLuc controls 4.03% (+/- 0.97%) (Figure 6b).

Life cell imaging was employed in order to examine the biology of the observed morphological changes in SOX2-depleted glioma cells. For the analysis we used U343 glioma cells which developed membrane protrusions accompanied by contractions of the cell body 5 days after transduction of the vector encoding shSOX2 #2378 and EGFP (Figure 6c). The cells further contracted and then developed larger protrusions until a fully rounded morphology was reached (Figure 6d, and additional file 2). Interestingly, fully rounded cells started to stretch and performed worm-like movements (Figure 6e, additional files 3, and 4). All analyzed shLuc control cells so far did not exhibit such remarkable alterations in cell morphology.

**Figure 7:**
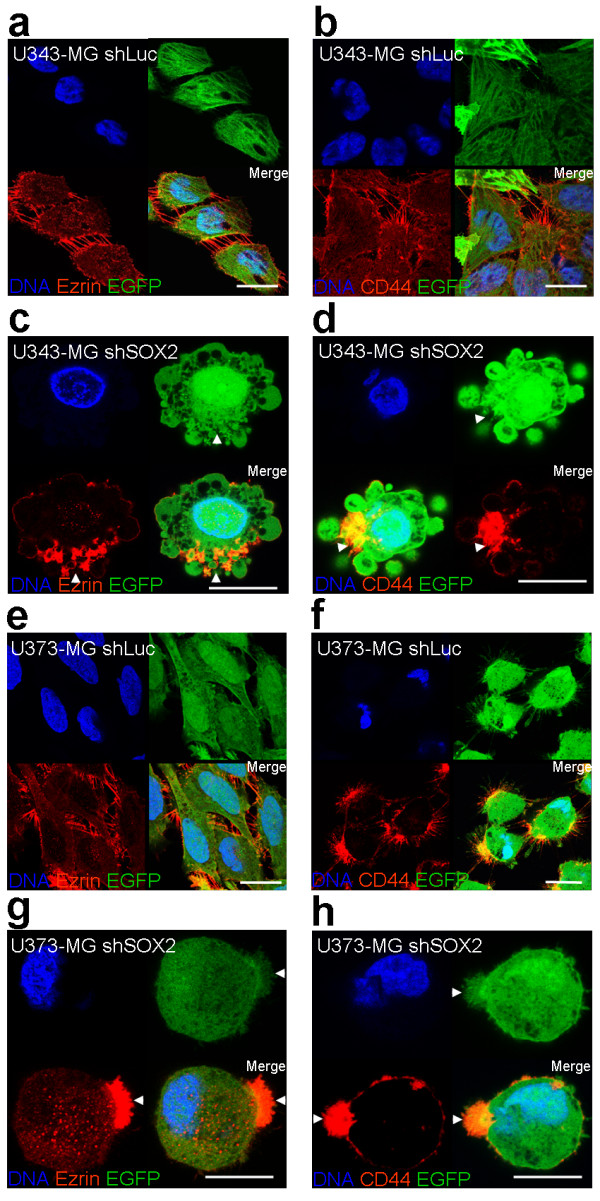
**U343-MG and U373-MG cells with knockdown of SOX2 develop a single membrane protrusion containing ezrin and CD44**. U343-MG and U343-MG glioma cells were transduced with retroviral vectors encoding EGFP and the indicated shRNA. Five days after transduction cells were fixed and stained with primary ezrin, CD44 antibodies and secondary Cy3-coupled antibodies as depicted in the images. DNA counterstaining was performed using Hoechst 33342. **a, b: **U343-MG cells transduced with shLuc control vector. **c, d: **U343-MG cells transduced with shSOX2 #sh2378. **e, f: **U373-MG cells transduced with shLuc control vector. **g, h: **U373-MG cells transduced with shSOX2 #sh2378. Note in **c: **and **g: **the development of a single cell protrusion containing ezrin and as shown in **d **and **h **the appearance of CD44 in single protrusions in cells with knockdown of SOX2. Magnification bars, in a, b, e, f: 20 μm; in c, d, g, h: 10 μm.

To further corroborate a possible association of the SOX2 RNAi-induced phenotype to the proteolysis-independent amoeboid migration mode we focused on the marker proteins CD44 and ezrin. Amoeboid migrating cells display an ezrin pole at the site of the membrane which faces the direction of movement [34-36]. The cytoskeletal protein ezrin connects actin filaments to the plasma membrane protein CD44 [37] and thereby links the mechanical force of the cytoskeleton to extracellular matrices (ECM) containing hyaluronic acid, a described ligand for CD44 [38]. In order to identify the isoforms of CD44 we performed Western blot analysis and identified the standard CD44 isoform (CD44s) in U343-MG and U373-MG cells (see additional file 5). Then we used confocal laser scan microscopy to investigate in detail the intracellular localization of CD44 and ezrin. In shLuc-U343-MG control cells ezrin and CD44 were concentrated around the cell body in long fiber-like protrusions (Figure 7a, b). In shLuc-U373-MG cells both proteins were seen in similar structures connecting the cell to the ground of the culture dish (Figure 7e, f). In contrast, in U343-MG and U373-MG cells with knockdown of SOX2 both proteins were found to be reorganized and were concentrated at a distinct pole emerging from the cell body (Figure 7c, d and 7g, h). Additional double immunofluorescence analyses using cells transduced with retroviral vectors in which the EGFP was substituted for a puromycin resistance cassette confirmed colocalization of CD44 and ezrin in the protrusions (see additional file 5).

**Figure 8:**
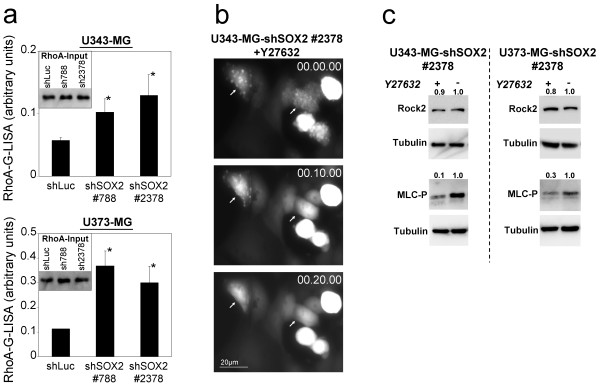
**SOX2-RNAi-induced membrane protrusions are associated with increased RhoA activity and are repressed by blocking ROCK**. **a: **RhoA-G-Lisa of U343-MG and U373-MG cells with knockdown of SOX2 compared to shLuc control cells. Protein lysates were prepared and subjected to RhoA-G-LISA. Inlays: RhoA-input in G-LISA. *p < 0.05 when cells with knockdown of SOX2 were compared to the shLuc controls. **b: **Inhibition of ROCK signaling downstream of RhoA with the small chemical compound Y27632 completely abolish membrane blebbing in U343-MG with knockdown of SOX2. The inhibitor was added at time 00.00.00 (hh:mm:ss). **c: **Western blot analysis confirms ROCK2 expression in U343-MG and U373-MG cells with knock down of SOX2 which is not affected by treatment with Y27632. The inhibition of ROCK led to diminished levels of phosphorylated myosin light chain (MLC-P). Equal loading of the filters was confirmed by stripping the filters and probing with tubulin antibodies. Densitometric analyses of Western blot signals normalized to tubulin signals are depicted.

In order to further prove the development of the amoeboid-like phenotype we focused on the RhoA/ROCK-pathway which is essential for amoeboid migration [34,39]. We did not detect ROCK1 protein (data not shown) whereas ROCK2 and RhoA were expressed in U343-MG and U373-MG cells (Figure 8a, c). In its active GTP-bound form, the GTPase RhoA activates ROCK2 which regulate contractile actin/myosin II elements [39].

A RhoA G-LISA was used to determine the levels of RhoA-GTP. To investigate RhoA regulation in U343-MG and U373-MG wild type cells, protein lysates of serum-starved cells and of cells subsequently treated with lysophosphatic acid (LPA), a common activator of RhoA, were included in the experiments. The analysis of these controls confirmed an intact RhoA regulation of U343-MG and U373-MG wild type cells (data not shown). When testing U343-MG and U373-MG cells with knockdown of SOX2, a significant increase of RhoA-GTP was observed when compared to the shLuc controls (Figure 8a). Hence, RNAi of SOX2 induces an increased RhoA signaling in U343-MG and U373-MG glioma cells.

Since increased RhoA-activation should lead to ROCK activation we sought to confirm the RhoA/ROCK activation on a functional level. We therefore utilized the compound Y27632, which is known to specifically inhibit ROCK proteins [40] and monitored its effects on SOX2-depleted U343-MG and U373-MG glioma cells using life cell imaging. As anticipated the dynamic membrane alterations of SOX2-depleted cells were abolished in both cell lines within a few minutes (Figure 8b, and see additional file 6). To further corroborate that ROCK2 was inactivated by Y27632 we analyzed the activation status of the myosin light chain (MLC). Phosphorylation of MLC (MLC-P) of myosin II at serine 19 induces its interaction with actin, which thereby activates myosin ATPase resulting in enhanced cell contractility [41]. It has been reported that besides the Ca^2+^-dependent myosin light chain kinase (MLCK) also ROCK kinases phosphorylate MLC at serine 19 [41]. In fact, we revealed that treatment with the ROCK inhibitor Y27632 led to a decrease in MLC-P levels (Figure 8c) which indicates that ROCK2 is involved in the development of the membrane protrusions occurring in the U343-MG and U373-MG glioma cells with knockdown of SOX2.

To verify that SOX2-depleted cells were motile, we employed an organotypic brain slice migration assay. Fresh quarters of mouse brains soaked with medium containing FCS were incubated with serum starved shLuc-U373-MG and shSOX2-2378-U373-MG cells, respectively. After 36 hours the shSOX2-transduced and the control cells had both invaded into the mouse brain tissue. Due to the experimental setting, it was not possible to precisely quantify, whether cells with knockdown of SOX2 or shLuc control cells have an improved migration. However, it became clear that the glioma cells with knockdown of SOX2 had the capacity to migrate into the brain tissue (see additional file 7).

### Knockdown of SOX2 increases spreading of orthopically transplanted U343-MG glioma cells and decreases survival of mice

**Figure 9:**
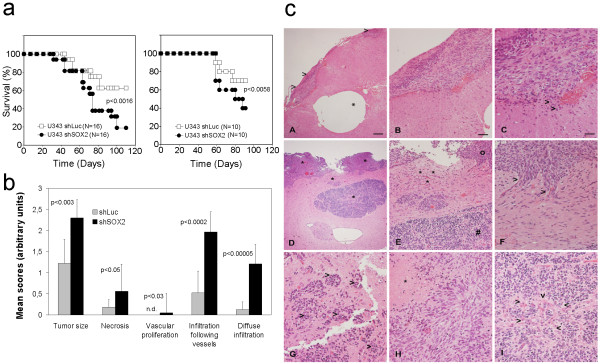
**U343-MG xenografts with knockdown of SOX2 are tumorigenic and show a increased spreading in the mouse brain**. **a: **Kaplan-Meier curves showing the survival of mice transplanted with 5 × 10^5 ^U343-MG cells with knock down of SOX2 and transduced with shLuc, respectively. Two independent experiments are depicted, which show a significantly reduced survival of mice transplanted with SOX2 depleted U343-MG cells. Number of animals for each experiment and p values for logRank-test are depicted. **b: **Semi-quantitative analysis of tumor size and specific traits of tumor spreading in the brain. Glioma cells with knockdown of SOX2 shows strong increased infiltration following vessels and diffuse infiltration into the normal brain parenchyma. Bars indicate standard deviation. The p values for student's t-Test are shown. **c: **Representative images of U343-MG gliomas in mouse brain. **A-C: **U343-MG tumors transduced with shLuc; **D-I: **U343-MG tumors transduced with shSOX #2378: **A: **Small superficially growing shLuc tumor (arrow heads) despite an intracerebral tumor cell reservoir (asterisk). **B, C: **Magnifications of the tumor area shows a compact growth pattern with clearly visible tumor borders and minimal single cell infiltration (arrowheads). **D: **U343-MG tumors with knockdown of SOX2 showing multilocular intracerebral growth (asterisks). **E: **Magnification of the tumor area showing two distinct tumor populations with spindle cell morphology and smaller rounded nuclei (#). Asterisks mark an area with intense single cell infiltration. **F: **Further magnification of the same area reveals tumor cells with spindle cell morphology growing along performed perivascular spaces (arrowheads). **G: **U343-MG cells with knock down of SOX2 growing at perivacsular spaces (arrowheads). **H: **Necrosis in U343-MG tumor with knockdown of SOX2 (asterisk). **I: **Area of U343-MG tumor with knockdown of SOX2 with small rounded nuclear morphology and glomeruloid vascular endothelium proliferation (arrowheads). Magnification bars for A and D: 160 μm; B and E: 80 μm; C, F, G, and I: 40 μm. n.d.: not detected.

In order to test the tumor initiation ability and the migratory phenotype of U343-MG cells with knockdown of SOX2 *in vivo *we stereotactically transplanted 5 × 10^5 ^glioma cells 5 days after transduction of shSOX2 #2378- and shLuc-vectors, respectively. Although stereotactically injected into the right hemisphere the U343-MG gliomas mostly grew along the surface of the brain. That this phenomenon was due to technical difficulties was excluded by parallel stereotactical injections of U87-MG cells, which were found to grow exclusively intra-parenchymal at the site of injection (data not shown). We conclude that the U343-MG cells exhibit a distinct growth pattern when compared to standard cell lines used for orthotopic xenografts such as U87-MG or U251-MG. Yet, we observed a significant reduced survival of mice transplanted with U343-MG cells transduced with shSOX2 #2378 when compared to controls (Figure 9a). In line with the survival data the U343-MG tumors with knockdown of SOX2 were larger. These tumors also showed a significantly increased infiltration following blood vessels but also significantly augmented diffuse infiltration into the brain parenchyma when compared to shLuc-transduced controls (Figure 9b, c) indicating an increased migratory capacity in the brain.

## Discussion

### SOX2 is involved in regulation of proliferation and differentiation status of U343-MG and U373-MG glioma cells

In our previous study we detected the stem cell marker and transcription factor SOX2 in glioblastoma tissue and glioblastoma cell lines [8] and hypothesized that it might represent an ideal target for an experimental RNAi approach for treatment of malignant gliomas. In fact, we found that RNAi of SOX2 attenuated proliferation of U343-MG and U373-MG glioma cells. Yet, cells with knockdown of SOX2 remained viable for weeks in cell culture, an observation which also has been reported by Gangemi et al. for glioblastoma tumor-initiating cells (TICs) with knockdown of SOX2 [24]. On the other hand, the same study revealed that the knockdown of SOX2 in glioblastoma TICs led to a moderate but sustained decrease in cell proliferation which was associated by a loss of Ki67 proliferation marker expression. Yet, the molecular mechanisms underlying the observed growth inhibition remained elusive and it was hypothesized that the loss of proliferative capacity was linked to some kind of differentiation of the glioblastoma TICs. In our study, we show for the first time that SOX2 knockdown led to an attenuated S-phase entry in malignant glioma cells which was caused by diminished levels of cyclinD1 and a concomitant decrease in the amount of phosphorylated RB protein. Recently, the same effect after SOX2 knockdown has been reported in MCF7 breast carcinoma cells. Here, a decrease in proliferation of MCF7 cells after SOX2-RNAi was demonstrated to be due to diminished cyclinD1 expression levels whereas ectopic overexpression of SOX2 appeared to facilitate the G_1 _to S_1 _transition. Noteworthy, the same study revealed that the cyclinD1 promoter region contains a functional SOX2 canonical binding site [26]. Hence, we suggest that RNAi of SOX2 in U343-MG and U373-MG glioma cells impairs cyclinD1 transcription and affects proliferation in the same way.

Interestingly, upon knockdown of SOX2 the expression of the stem cell markers Nestin and Oct4 was decreased in U343-MG and U373-MG glioma cells supporting the view that SOX2 preserves a more undifferentiated cell phenotype as previously observed in neural stem cells [12]. In parallel, an increased TCF/LEF-signaling was noted. These results suggest that SOX2 and TCF/LEF-signaling balances stem cell-like characteristics and differentiation in U343-MG and U373-MG glioma cells. So far we do not know the molecular mechanism how loss of SOX2 affects TCF/LEF-signaling. Yet, a recent report demonstrated that SOX2 maintains self renewal of murine tumor-initiating osteosarcoma cells which were driven into osteogenic differentiation through increased basal TCF/LEF-signaling after knockdown of SOX2 and lost proliferative capacity [29]. Interestingly, the same study showed that an increase in wnt-mediated TCF/LEF-signaling led to a decrease in endogenous SOX2 expression. We suggest that SOX2 expression in U343-MG and U343-MG glioma cells might inhibit TCF/LEF-signaling in the same way keeping the glioma cells in a more undifferentiated stem cell-like state. Although not proven so far, it also appears conceivable that the observed loss of intercellular adherence and loss of focal adhesion after RNAi of SOX2 in U343-MG and U373-MG glioma cells directly affects TCF/LEF-signaling through elevated levels of cytosolic β-Catenin.

However, the question whether increased TCF/LEF-signaling might affect the migratory capacity of the U343-MG and U373-MG glioma cells and therefore potentially increases tumorigenicity (described in the following paragraph) remains to be investigated.

### SOX2 RNAi impairs proteolysis-dependent invasion and induces an amoeboid-like phenotype

To our knowledge the migratory capacity of SOX2 silenced malignant glioma cells has not been investigated so far. We report for the first time, that knockdown of SOX2 impairs the invasive proteolysis-dependent migration of glioma cells. Furthermore, we demonstrated that loss of invasive proteolysis-dependent migration of U343-MG and U373-MG cells was associated with decreased FAK-signaling and diminished HEF1/NEDD9 protein levels. Also, the expression levels of pro-MMP1 and pro-MMP2, which have been reported to be regulated by Ets-1 (v-ets erythroblastosis virus oncogene homolog 1) transcription factor [42,43] downstream of SOX2 [44,45] were diminished after SOX2-RNAi.

Remarkably, U343-MG and U373-MG glioma cells with knockdown of SOX2 were able to compensate the loss of invasive proteolysis-dependent migration capacity by acquiring an amoeboid-like migration modus [46]. To our knowledge, only mesenchymal migration has been reported for glioma cells so far [47]. The shift to the amoeboid-like migration mode was accompanied by a reorganization of the cytoskeleton. We identified a loss of actin stress fibers and the development of a pronounced cortical actin network with concomitant formation of a cell pole containing ezrin and CD44. CD44 has been described to link the actin cytoskeleton via ezrin to hyaluronic acid (HA) [48,49]. HA is the principal glycosaminglycan found in the extracellular matrix (ECM) of the brain and is distributed in white matter fiber tracts, which is proposed to be the most frequent route of glioma dissemination [50].

In line with previous reports describing amoeboid migration, we detected an increased RhoA/ROCK2 signaling in U343-MG and U373-MG cells with knockdown of SOX2 [33,34]. That RhoA/ROCK2 signaling was essential for this kind of migration was verified by blocking ROCK2 downstream of RhoA by using the inhibitor Y27632, which completely abolished the development of an amoeboid-like phenotype.

So far we have not investigated the molecular mechanisms linking SOX2 and RhoA/ROCK2. However, a recent report indicates a role of cyclinD1 in inhibiting RhoA/ROCK expression and signaling in mouse embryonic fibroblasts whereas a cyclinD1 knock out increased RhoA/ROCK-signaling [51]. Since we observed that SOX2 depletion led to a decrease in cyclinD1 expression we suggest that the same mechanism might be involved in the increased RhoA/ROCK2 signaling in U343-MG and U373-MG glioma cells.

That the acquired amoeboid-like migration mode was functional could be demonstrated by transwell assays using membranes lacking ECM. Furthermore, the SOX2-depleted cells were able to invade into mouse brain tissue often showing a typical morphology bearing membrane blebs. Strikingly, when we tested U343-MG glioma cells with knockdown of SOX2 in orthotopic xenografts we observed a significant decreased survival of these mice when compared to controls. Furthermore, histological analysis of the tumors demonstrated that significantly more SOX2-depleted U343-MG cells spread along vessels or diffusely spread into the normal brain tissue. Our results contradict the study from Gangemi et al. which demonstrates that RNAi of SOX2 reduces the tumorigenicity of glioblastoma tumor-initiating cells [24]. Our study furthermore contradicts reports which show that ectopic overexpression of SOX2 enhances tumorigenicity of prostate cancer cells [52] and of breast cancer cells [53] but is in line with reports indicating a tumor-suppressive function of SOX2 in gastric cancer cells lines [22,54]. So far we cannot resolve the observed discrepancies and it remained to be clarified whether the genetic background of the cells, differentially expressed molecular partners of SOX2 (i.e. Oct3, Nanog), the long term adaptation of U343-MG and U373-MG in cell culture or technical differences account for the observed effects following RNAi of SOX2. Nonetheless, in our study we demonstrate for the first time a role of SOX2 in migration of glioma cells. Noteworthy, in our experiments we showed that SOX2 seems not to be fundamental for the maintenance of U343-MG and U373-MG glioma cell proliferation.

## Conclusions

In summary, we have confirmed SOX2 as important factor involved in the preservation of a less differentiated glioma cell phenotype. Yet, our studies revealed that SOX2 regulates cell-matrix interaction and invasive proteolysis-dependent invasion of U343-MG and U373-MG cells. Although long term GBM cell lines have their limitations since they might differ in some aspects from primary tumor cells, caution is advised when setting up treatment strategies targeting SOX2 in glioblastoma patients, since a decrease in invasion proteolysis-dependent migration capacity might to some extent be compensated by a switch to an amoeboid-like migration which might affect survival of patients. Further investigations using the SOX2-depleted glioblastoma cells and primary GBM cell preparations, respectively, are warranted which should give further insights of the role of SOX2 in glioma cell biology.

## Methods

### Cell culture methods

U373-MG is a glioblastoma/astrocytoma derived cell line; U343-MG is a glioblastoma-derived cell line. The glioma cells were cultured on poly-L-lysine coated plastic ware and in Basal Minimal Eagle medium (BME, Invitrogen, Eggenstein, Germany) supplemented with 2 mM L-glutamine and 1% non-essential amino acids (Biochrom, Berlin, Germany). 293T are human embryonic kidney cells. They were cultured in Dulbecco's modified Eagle medium containing 4.5 g/l glucose (PAA Laboratories, Pasching, Austria). The medium was supplemented with 10% heat inactivated fetal calf serum, 100 U/ml penicillin and 100 μg/ml streptomycin (both Invitrogen). The proliferation of transduced glioma cells was determined by counting total cell numbers using a Neubauer microscope counting chamber. In brief, 10^5 ^cells were plated in triplicates at day 0 in 6 well culture plates and viable cells were counted after 3, 5 and 7 days. Long term survival of transduced glioma cells was tested by plating 1000 cells/dish. After three weeks U343-MG and U373-MG cells were stained with Giemsa, and the number of clones was quantified. The percentage of cells displaying membrane alterations ("blebs") after transduction with retroviral vectors was detected and quantified by counting at least 3 randomly selected areas with at least 200 EGFP-positive cells using a Zeiss Axiovert 135 fluorescence microscope (Zeiss AG, Jena, Germany) at 400× magnification. All experiments were performed in triplicates and repeated at least for 2 times with similar results. Statistical analysis was performed with student's T test.

### SOX2-shRNA retroviral vectors and transduction

For the transduction of DNA-sequences encoding shRNA-molecules we used the self inactivating retroviral Moloney murine leukemia virus backbone pRVH-1. This vector contains a H1 polymerase III promoter for the expression of shRNA molecules in reverse orientation. pRVH1 was digested with EcoRI and NotI and ligated with an CMV immediate early promoter and EGFP containing appropriate restriction sites, resulting in the vector pRVH-N1-EGFP. 9 different SOX2 target sequences were identified using an algorithm provided by Ambion Inc. http://www.ambion.com, synthesized (Eurofins MWG Biotech, Ebersberg, Germany) and after annealing of the upper and bottom strands were ligated into the BglII/SalI-restrictions sites of pRVH-N1-EGFP. After testing, the following most effective shSOX2 hairpin sequences were used in our experiments: 788 upper: 5'-gatccccGAAGGATAAGTACACGCTGTTCAAGAGACAGCGTGTACTTATCCTTCTTTTTT*gg*c-3', 788 bottom: 5'-tcgagccAAAAAAGAAGGATAAGTACACGCTG TCTCTTGAACAGCGTGTACTTATCCTTCggg-3', 2378 upper: 5'-gatccccCTGC CGAGAATCCATGTATATCTCGAGATATACATGGATTCTCGGCAGTTTTT*gg*c-3', 2378 bottom: 5'-tcgagccAAAAACTGCCGAGAATCCATGTATATCTCGA GATATACATGGATTCTCGGCAGggg-3'. As control we included a previously described RNA hairpin against luciferase [55]. Retroviral particles were generated as described previously [56]. Briefly, 293T cells were cotransfected with an expression construct for gag-pol (pHIT60), the MoMuLV-based retroviral vectors and the vesicular stomatitis virus G-protein (pMD.G2). Viral supernatants were harvested 48 and 72 h after transfection. 10^5 ^target cells were plated in 30 mm dishes a day before transduction and were transduced with retroviral supernatants at a multiplicity of infection (MOI) of 20. Transduction experiments were freshly performed for each single experiment in triplicates for shSOX2 and shLuc vectors and used for the analyses. Transduction efficiencies usually were in the range of 95%-99%. If not otherwise indicated, the transduced cells were cultivated for five days in BME medium with supplements and then were used for the experiments.

### Life cell imaging

Cells were cultured and imaged in 2-well chamberslides (Nalge Nunc, Rochester, USA) using an inverse AF 6000LX microscope (Leica, Wetzlar, Germany) with a humidified chamber at 37°C and 5% CO_2_. Five days after transduction with retroviral vectors encoding shSOX2 #2378 and shLuc, respectively, pictures of cells were taken every 5 or 10 minutes. For ROCK inhibition experiments 16 μl of a 10 μM Y27632 solution in H_2_Odd were added to the cells per ml culture medium. The experiments were repeated four times with similar results.

### Phalloidin-TRITC staining and indirect immunofluorescence analysis

Transduced cells on poly-L-Lysine mounted glass slides were fixated for 20 min with 4% paraformaldhyde (PFA) in PBS. Fixated cells were treated with ice cold permeabilization solution (0.1% sodium citrate in PBS, 0.1% Triton X-100) for 5 min. Filamentous actin was stained with phalloidin-TRITC (8 μM) (Sigma) for 1 h. Confocal laser scan analysis was performed using Leica SP5 inverse microscope (Wetzlar, Germany). Thickness of cortical actin filaments of cells with SOX2 knockdown and with affected morphology was compared to shLuc control cells using the Leica Application Suite Software 2.2.0. For indirect immunofluorescence analyses following primary antibodies were used: Anti-h-CD44 (mouse monoclonal; 1:100; BD Biosc.), anti-h-ezrin (mouse monoclonal; 1:100; BD Biosciences, Franklin Lakes, USA), anti-h-OCT4 (rabbit polyclonal; 1:100; Abcam, Cambridge, USA), anti-h-SOX2 (goat polyclonal; 1:100; R&D Systems, Minneapolis, USA). Cells were washed 3 times with PBS + 0.1% BSA and subsequently incubated with species specific secondary antibodies (Cy3 conjugated; 1:100; Jackson IR, West Grove, PA) for 1 hour. For colocalization studies of CD44 and Ezrin we used non-fixed transduced cells which were stained using anti-h-ezrin and secondary FITC-conjugated secondary antibody followed by staining with PE-labeled anti CD44 (mouse monoclonal, 1:100, BD Biosciences). Finally, cells were washed three times in PBS/0.1% BSA and once in double distilled water, before being examined by confocal laser scanning microscopy (Leica SP5 inverse MP).

### Western blot analysis

Total cell lysates were prepared using 2× Laemmli-protein-sample-buffer (Sigma). Samples were subsequently cooked for 5 min and placed in an ultrasonic bath for 15 min. Equal amounts of proteins was separated in SDS-polyacrylamide gels and blotted onto PVDF membranes. After blocking the membranes were incubated with the following primary antibodies for 1 hour: anti-cyclinD1 (rabbit polyclonal; 1:500; Santa Cruz Biotech, Heidelberg, Germany), anti-cyclinE (rabbit polyclonal; 1:500; Santa Cruz), anti-h-FAK (mouse monoclonal; 1:1000; BD Biosciences), anti-h-FAK(pY397) (mouse monoclonal; 1:1000; BD Biosciences), anti-h-GFAP (mouse monoclonal; 1:1000; Chemicon, Chandlers Ford, UK), anti-h-NEDD9/HEF1 (mouse monoclonal; 1:1000; Abcam), anti-h-MMP1 (mouse monoclonal; 1 μg/ml; R&D Systems), anti-h-MMP2 (mouse monoclonal; 1 μg/ml; R&D Systems), anti-h-p21 (mouse monoclonal; 2 μg/ml; R&D Systems), anti-h-RB (mouse monoclonal; 1:750; Abcam), anti-h-phosphoRB (mouse monoclonal; 0.75 μg/ml; Sigma), anti-h-SOX2 (goat polyclonal; 1:1000; R&D Systems), anti-β-Catenin (mouse monoclonal, BD Biosciences; 1:1000), anti-ROCK1 (mouse monoclonal, Santa Cruz; 1:1000), anti-ROCK2 (rabbit polyclonal, Santa Cruz; 1:500), anti phospho-Serine19 myosin light chain (MLC-P) (mouse monoclonal, Cell Signaling Technologies, Danvers, USA, 1:500) anti-h-αTubulin (mouse monoclonal; 1:1000; Sigma). Induction of apoptosis was analyzed by using an anti Caspase 3 antibody (mouse monoclonal; 1:1000; Cell Signaling Technology). After incubation with the primary antibody the membranes were washed three times with TBS-TT and once 10 min with TBS-buffer. Subsequently the membranes were incubated for 1 hour with appropriate species specific secondary antibodies conjugated with HRP (1:1000; Dako, Glostrup, Denmark). After washing the blots were imaged using ECL-detection solution (GE Healthcare, Freiburg, Germany) and a LAS3000 device.

### RhoA activation assay

RhoA-GTP was measured using a RhoA-G-LISA™ (Cytoskeleton Inc., Denver, USA) according to the instructions of the provider. As a negative control fraction wild type cells were starved in serum free medium to completely deactivate RhoA [57]. As positive control for fully activated RhoA signaling type cells were treated with lysophosphatic acid (LPA), a common RhoA activator [58]. Probes were measured in a Tecan ELISA reader at 490 nm. shLuc-transduced cells were compared to cells transduced with SOX2 #sh788 and SOX2 #sh2378, respectively. The experiment was performed in triplicates and repeated twice with similar results. Statistical analysis was performed with student's T test.

### Flow cytometry for cell cycle analysis and apoptosis

Analysis of the cell cycle and apoptosis was determined by flow cytometry of propidium iodide-stained cells using Cell Quest Software (BD, Heidelberg, Germany). Briefly, adherent cells and cells in the cell culture supernatant were collected, washed in PBS and fixed in 70% (v/v) ethanol. After centrifugation of the cells at 600 g at 20°C for 10 min the cell pellet was suspended in 0.5 ml DNA-extraction buffer (4 mM citric acid in 0.2 M Na_2_HPO_4_; pH 7.8). After 5 min incubation at room temperature the cells were spun down at 600 g. The cells were then washed once with PBS, followed by incubation in PBS containing 40 μg/ml propidium iodide (Sigma, Taufkirchen, Germany) and 200 μg/ml RNase A (Sigma, Dreieich, Germany) for 1 h at room temperature in the dark. Stained nuclei were then analyzed using a Becton Dickinson FACScan (BD) with at least 10,000 events/determination. The experiments were performed in triplicates and repeated at least twice. Statistical analysis was performed with student's T test.

### Migration and invasion assays

The invasion activity of SOX2-depleted cells was measured by Boyden-chamber assay using BD BioCoat Matrigel Invasion Chambers™ (BD Biosciences) which consists of a membrane with 8 μm pores and a basement matrix, as recommended by the supplier. 10,000 cells were serum-starved for 24 h and plated with serum-free BME medium in the insert chambers. The lower chambers were filled with DMEM with 10% FCS. After 24 h incubation the degraded matrigel was scrapped off and the membranes containing the invaded cells were removed. The EGFP-positive cells that had invaded through the Matrigel and pores to the other side of the membrane were fixed using 4% paraformaldehyde, and counted using a Zeiss Axiovert 135 fluorescence microscope. To determine the invasion index we employed uncoated Boyden-chambers (BD BioCoat Companion plates (BD Biosciences)) having 8 μm pores. The calculation of the invasion index was made by the formula cell number of invading cells through matrix/cell number of cells on control filter (BD Companion plate). Due to the lack of adherence to the filter, cells having an amoeboid-like phenotype could be measured in the lower chambers of Companion plates. The calculation of the amoeboid migration index was made by the formula cell number in the lower well/cell number on control filter. All migration experiments were performed in triplicates and repeated twice. Statistical analysis was performed with student's T test. For the brain tissue invasion assay freshly prepared roughly 8 mm^3 ^cubes of mouse brains were incubated for 5 hours in BME medium with 10% FCS in a 48 well plate. Thereafter the brain pieces were transferred into clean wells and washed with sterile PBS and transferred into wells containing suspensions of 10,000 serum-starved SOX2-depleted cells and control cells, respectively, in serum free BME medium. After 36 h the brain cubes were transferred into clean wells, washed and covered with serum free BME medium. Subsequently the samples were analyzed and imaged using a Zeiss Axiovert 135 fluorescence microscope. The experiments were repeated twice with similar results.

### TCF/LEF-luciferase reporter gene assay

U343-MG and U373-MG glioma cells were transduced with lentiviral particles of Lenti TCF/LEF luciferase reporter at a MOI of 25 (SABiosciences/BIOMOL GmbH, Hamburg, Germany). After selection with puromycin the cells were transduced with retroviral pRVH1-sh2378-EGFP and empty vector, respectively. 5 days after transduction the activity of the TCF/LEF-element was measured with 50,000 cells/well in triplicates using the Luciferase Assay System (Promega, Mannheim, Germany) and an Orion Microplate Luminometer (Berthold Technologies, Bad Wildbad, Germany). The luciferase mediated light emission was normalized to the expression level of the co-transduced reporter EGFP as determined in Western blot analysis. The experiments were performed in quadruplicates and at least repeated twice. Statistical analysis was performed with student's T test.

### Mouse xenograft assays

9 weeks old NMRI^nu/nu ^mice were obtained from the animal facility of the University of Dresden. Mice were held under standardized pathogen-free conditions with *ad libitum *access to food and water. Experiments were approved by the responsible local authorities according to the German Animal Protection Law. 5 × 10^5 ^U343-MG cells resuspended in 10 μl PBS were used for stereotactical injections in the right brain hemisphere. In total, survival of 26 mice transplanted with U343-MG cells with knockdown of SOX2 and survival of 26 mice transplanted with shLuc-transduced control cells was investigated in two independent experiments. Mice were killed when neurological symptoms became apparent. 12 brains transplanted with U343-MG cells with knockdown of SOX2 and 9 brains transplanted with shLuc-transduced U343-MG cells were randomly selected and were subjected to standard histology. Whole brains of mice were fixed in buffered 4% formalin (pH 7), cut into 3-4 frontal slices per brain, dehydrated and embedded in Paraffin. 5 μm thick serial sections were cut and stained with hematoxylin and eosin (H&E) and then evaluated using a Zeiss Axioplan 2 microscope. A semi-quantitative scoring system using scores from 1-4 was used to calculate the size of the tumors and specific traits such as necrosis, endothelial proliferation, spreading along perivascular spaces or diffuse single cell spreading according to a scoring system described previously [59]. Briefly, score values were as follows: Tumor size: Score 1 = visible on surface and < 0.1 cm in diameter, Score 2 = up to 5-10% total volume of brain and 0.1-0.2 cm in diameter, Score 3 = up to 10-30% total volume of brain and > 0.2 cm in diameter, Score 4 = more than 50% of total volume of brain and > 1 cm in diameter; Necrosis: Score 1 = necrotic area < 0.1 cm in diameter, Score 2 = necrotic area up to 0.2 cm in diameter, Score 3 = necrotic areas > 0.2 cm or with multiple smaller areas; Vascular proliferation: Score 1 = suspicious endothelium or single vascular proliferation, Score 2 = glomeruloid vascular proliferation; Infiltration following vessels: Score 1 = tumor cells infiltration following up to two vessels, Score 2 = infiltration following three to seven vessels, Score 3 = Infiltrations following more than seven vessels; Diffuse infiltration: Score 1 = few suspicious cells, Score 2 = strongly increased cellularity (up to 50% increase), Score 3 = more than 50% increase in cellularity. Student's t-test and LogRank test were used for statistical analysis

## Competing interests

The authors declare that they have no competing interests.

## Authors' contributions

FO and NM carried out the RNAi experiments, the analysis of the phenotype and statistics. GS participated in the design and helped to draft the manuscript. SH constructed retroviral vectors used in this work and helped with the brain slice experiments. DM performed stereotactical injections into mice brains. KDG carried out the histology and interpretation of data. AT designed and coordinated the study, designed the shRNAs, and drafted the manuscript. All authors read and approved the final manuscript.

## Supplementary Material

Additional file 1 **This figure shows transduction efficiencies of retroviral shRNA-vectors and clonogenic long term survival of U343-MG and U373-MG cells with knock down of SOX2**. **a: **Representative FACS analysis showing EGFP marker gene expression of U343-MG and U373-MG glioma cells two days after transduction with the depicted retroviral vectors. Open histograms represent non transduced cells, black histograms depict transduced cells expressing EGFP. **b: **Clonogenic survival of U343-MG and U373-MG cells transduced with shSOX2 #2378 and shLuc control, respectively. The number of surviving clones was quantified. *p < 0.05 when U343-MG cells with knockdown of SOX2 were compared to the shLuc controls. Click here for file

Additional file 2 **Time lapse video imaging showing the development of membrane protrusion s in U343-MG cells with knockdown of SOX2**. This video file shows the development of membrane protrusions and amoeboid-like movements in U343-MG glioma cells after knockdown of SOX2. The file can be viewed using Windows Media Player or other standard media players. Click here for file

Additional file 3 **Time lapse video imaging showing blebbing and movement of U343-MG cells with knockdown of SOX2**. This video file shows a single U343-MG glioma cells after knockdown of SOX2 and the transition from blebbing to an amoeboid-like movement. In particular the development of a single protrusion in the direction of movement can be seen. The file can be viewed using Windows Media Player or other standard media players. Click here for file

Additional file 4 **Time lapse video imaging showing cell motility of U343-MG cells with knockdown of SOX2**. This video file shows the motility of a single U343-MG glioma cells after knockdown of SOX2. The file can be viewed using Windows Media Player or other standard media players. Click here for file

Additional file 5 **This figure shows expression of CD44s in U343-MG and U373-MG cells and its colocalization with ezrin in single membrane protrusions of cells with amoeboid appearance**. **a: **Western Blot showing expression of the standard isoform of CD44 (CD44s) in U343-MG and U373-MG glioma cells. **b: **Confocal laser scan microscopy showing the colocalization of CD44 and ezrin at membranes of shLuc-transduced cells and in single membrane protrusion in cells with knock down of SOX2 (arrowheads). Bars represent 10 mm. Click here for file

Additional file 6 **Time lapse video imaging showing that ROCK-inhibitor Y27632 blocks blebbing and membrane protrusions in U343-MG cells with knockdown of SOX2**. This video file shows U343-MG glioma cells after knockdown of SOX2 and ongoing membrane protrusions and retractions. After adding the ROCK inhibitor Y27632 the formation of the membrane protrusions (blebs) is blocked. The file can be viewed using Windows Media Player or other standard media players. Click here for file

Additional file 7 **This figure shows images of migrating U373-MG cells with knock down of SOX2 and of controls in organotypic brain tissue**. Brain tissue invasion assay showing infiltration of **a: **starved U343-MG and **c: **starved U373-MG cells transduced with shSOX2 #2378 and of **b: **starved shLuc-transduced U343-MG and of starved shLuc-transduced U373-MG control cells into murine brain tissue soaked with BME medium containing 10% fetal calf serum. **a: **Note the appearance of U343-MG and in **b: **U373-MG cells with knockdown of SOX2 and membrane protrusions. Cell displaying membrane protrusions are enlarged. U343-MG and U373-MG shLuc control cells normally displayed an elongated spindle-like morphology (see close up). Arrows depict the border of the brain tissues. Click here for file
