# Supplementary figures and images for: SOX2-RNAi attenuates S-phase entry and induces RhoA-dependent switch to protease-independent amoeboid migration in human glioma cells
Source: Mol Cancer. 2011 Nov 9;10:137. doi: 10.1186/1476-4598-10-137 (PMC3228695; doi:10.1186/1476-4598-10-137)

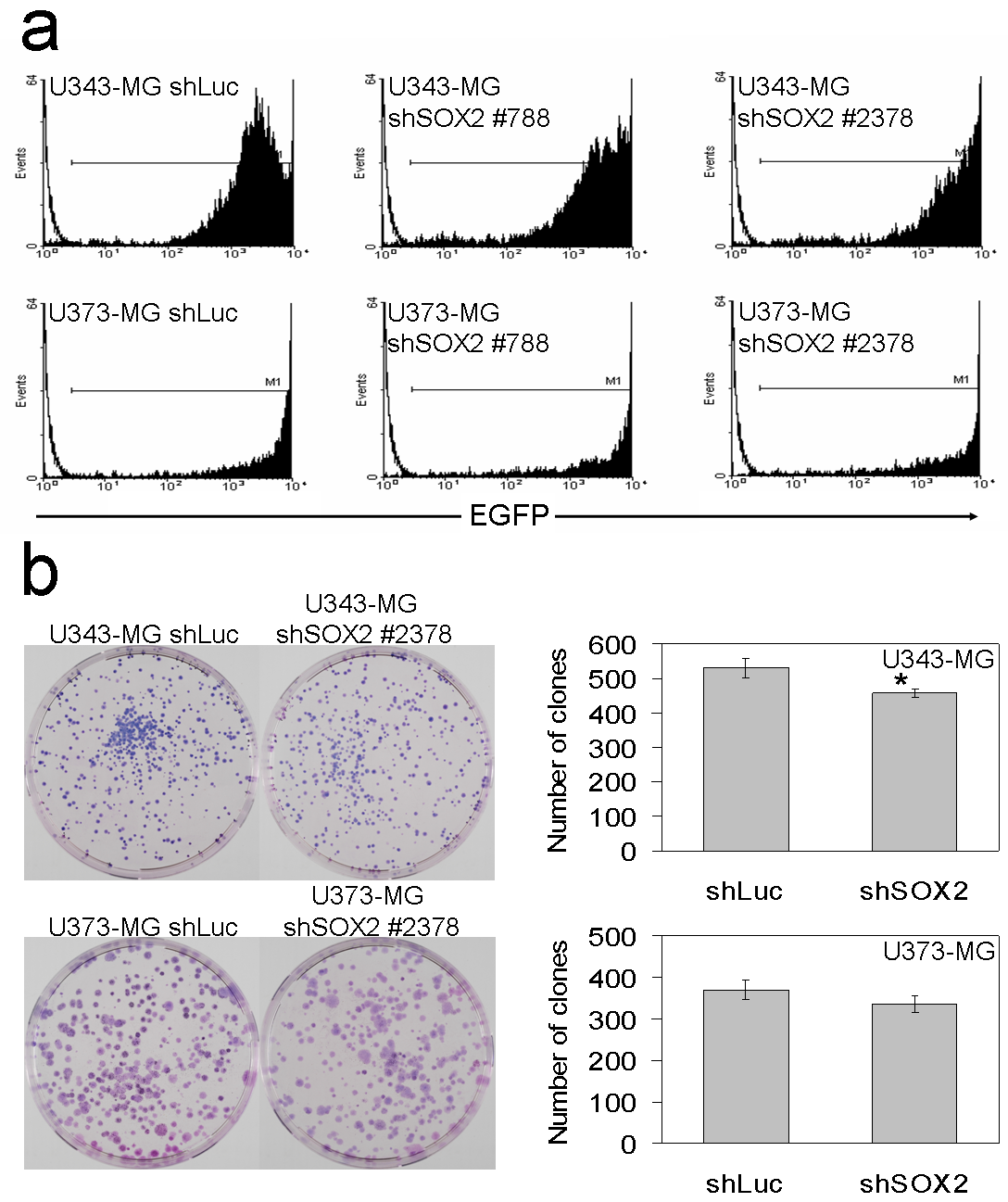

Supplement: Additional file 1 — This figure shows transduction efficiencies of retroviral shRNA-vectors and clonogenic long term survival of U343-MG and U373-MG cells with knock down of SOX2. a: Representative FACS analysis showing EGFP marker gene expression of U343-MG and U373-MG glioma cells two days after transduction with the depicted retroviral vectors. Open histograms represent non transduced cells, black histograms depict transduced cells expressing EGFP. b: Clonogenic survival of U343-MG and U373-MG cells transduced with shSOX2 #2378 and shLuc control, respectively. The number of surviving clones was quantified. *p < 0.05 when U343-MG cells with knockdown of SOX2 were compared to the shLuc controls. [file 1476-4598-10-137-S1.TIFF]

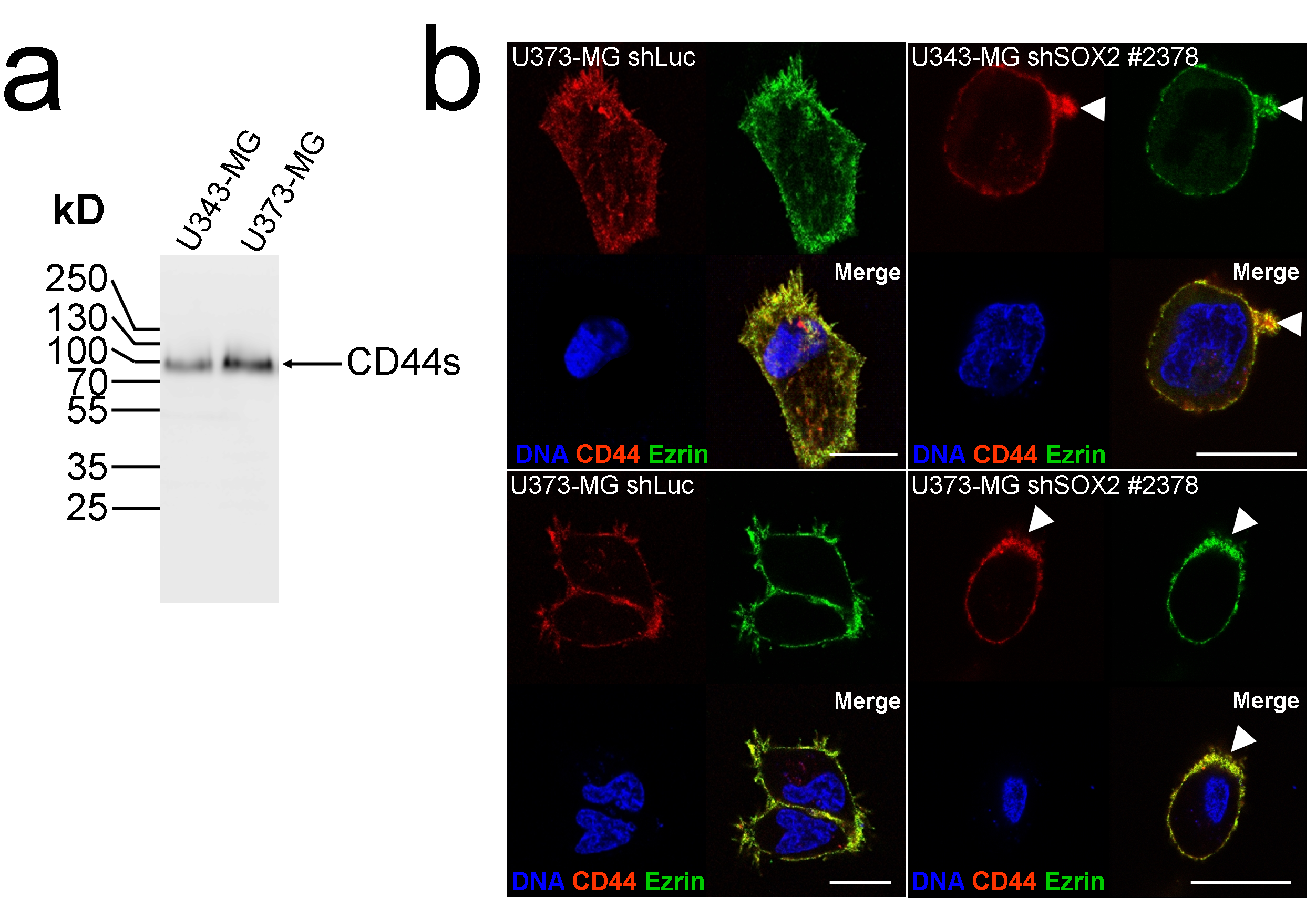

Supplement: Additional file 5 — This figure shows expression of CD44s in U343-MG and U373-MG cells and its colocalization with ezrin in single membrane protrusions of cells with amoeboid appearance. a: Western Blot showing expression of the standard isoform of CD44 (CD44s) in U343-MG and U373-MG glioma cells. b: Confocal laser scan microscopy showing the colocalization of CD44 and ezrin at membranes of shLuc-transduced cells and in single membrane protrusion in cells with knock down of SOX2 (arrowheads). Bars represent 10 mm. [file 1476-4598-10-137-S5.TIFF]

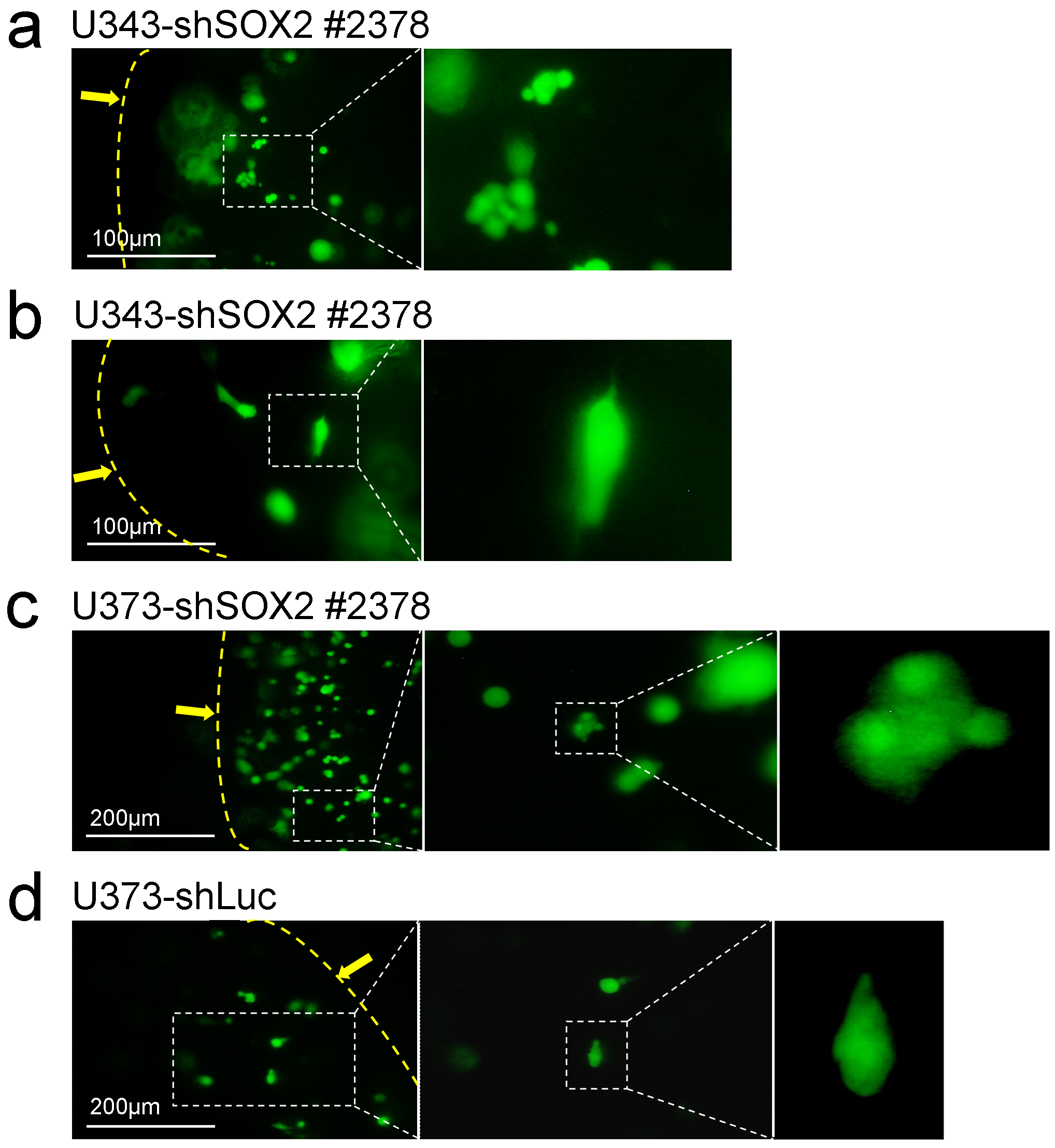

Supplement: Additional file 7 — This figure shows images of migrating U373-MG cells with knock down of SOX2 and of controls in organotypic brain tissue. Brain tissue invasion assay showing infiltration of a: starved U343-MG and c: starved U373-MG cells transduced with shSOX2 #2378 and of b: starved shLuc-transduced U343-MG and of starved shLuc-transduced U373-MG control cells into murine brain tissue soaked with BME medium containing 10% fetal calf serum. a: Note the appearance of U343-MG and in b: U373-MG cells with knockdown of SOX2 and membrane protrusions. Cell displaying membrane protrusions are enlarged. U343-MG and U373-MG shLuc control cells normally displayed an elongated spindle-like morphology (see close up). Arrows depict the border of the brain tissues. [file 1476-4598-10-137-S7.TIFF]
